# Sex-related variations in platelet reactivity in presence or absence of antiplatelet therapy

**DOI:** 10.1093/ehjcvp/pvaf034

**Published:** 2025-05-14

**Authors:** Mattia Galli, Sergio Terracina, Eleonora Schiera, Simone De Corci, Diego Sangiorgi, Massimo Mancone, Luigi Frati, Sebastiano Sciarretta, Dominick J Angiolillo, Fabio M Pulcinelli

**Affiliations:** Cardiovascular Department, Maria Cecilia Hospital, GVM Care & Research, Cotignola 48033, Italy; Department of Medical-Surgical Sciences and Biotechnologies, Sapienza University of Rome, Latina 04100, Italy; Department of Experimental Medicine, Sapienza University of Rome, Viale Regina Elena 324, Rome 00161, Italy; Department of Experimental Medicine, Sapienza University of Rome, Viale Regina Elena 324, Rome 00161, Italy; Department of Experimental Medicine, Sapienza University of Rome, Viale Regina Elena 324, Rome 00161, Italy; Cardiovascular Department, Maria Cecilia Hospital, GVM Care & Research, Cotignola 48033, Italy; Department of Clinical Internal, Anesthesiological and Cardiovascular Sciences, Sapienza University of Rome, Viale Regina Elena 324, Rome 00161, Italy; IRCCS NeuroMed, Pozzilli, Italy; Department of Medical-Surgical Sciences and Biotechnologies, Sapienza University of Rome, Latina 04100, Italy; IRCCS NeuroMed, Pozzilli, Italy; Division of Cardiology, University of Florida College of Medicine, Jacksonville, FL 32209, USA; Department of Experimental Medicine, Sapienza University of Rome, Viale Regina Elena 324, Rome 00161, Italy

**Keywords:** aspirin, Clopidogrel, Antiplatelet therapy, Sex, P2Y_12_ inhibitors

## Abstract

**Aims:**

Emerging evidence suggests sex-specific differences in platelet biology and clinical responses to antiplatelet agents. Light transmission aggregometry (LTA) represents the historical gold standard for the assessment of platelet reactivity but is influenced by pre-analytical and analytical variables. We analysed a large dataset of patients undergoing LTA using a standardized methodology to investigate the impact of sex on platelet reactivity with or without antiplatelet therapy.

**Methods and results:**

Between 2004 and 2022, 11,913 patients sequentially underwent LTA assessments following stimulation with adenosine diphosphate (ADP) (2 µM), collagen (2 µg/mL), arachidonic acid (AA, 0.5 mM), and epinephrine (10 µM). After applying study entry criteria, 5687 patients were included: 428 healthy volunteers (HV, *F* = 273; *M* = 155), 1055 controls (CTR; *F* = 725; *M* = 330), 3289 aspirin-treated patients (ASA; *F* = 2058; *M* = 1231), 430 clopidogrel-treated patients (CLOP; *F* = 272; *M* = 158), and 485 patients on dual antiplatelet therapy (DAPT; *F* = 166; *M* = 319). Within each group, results were analysed and compared between males and females.

Females exhibited significantly greater platelet reactivity in response to ADP compared to males in the HV (*P* = 0.004), CTR (*P* < 0.0001), ASA (*P* < 0.0001), and CLOP (*P* < 0.018) groups, but not in the DAPT group. Among aspirin-treated patients, females showed increased platelet reactivity (*P* < 0.0001) in response to collagen, compared with males.

**Conclusion:**

Females exhibit heightened baseline ADP-dependent platelet reactivity and a diminished response to aspirin and clopidogrel monotherapy compared to males.

## Introduction

Antiplatelet agents, such as aspirin and P2Y_12_ inhibitors (i.e. clopidogrel, prasugrel, and ticagrelor), used as monotherapy or in combination (dual antiplatelet therapy, DAPT), play a pivotal role in the primary and secondary prevention of atherosclerotic cardiovascular disease (ASCVD).^1^ However, their use is inherently associated with an increased risk of bleeding, which carries significant prognostic implications and, in certain circumstances, may outweigh their therapeutic benefits.^2^

Growing evidence highlights substantial variability in individual responses to antiplatelet therapy, which significantly influences the risk-benefit balance of these treatment.^3^ This variability in response to antiplatelet therapy is particularly pronounced in clopidogrel-treated patients, but may also occur with other antiplatelet agents.^4^ Patients with poor responsiveness to antiplatelet therapy, resulting in high platelet reactivity (HPR), face an increased risk of thrombotic events compared to responders.^3^ Conversely, patients with heightened responsiveness, leading to low platelet reactivity (LPR), are at greater risk of bleeding complications without additional reductions in thrombotic risk.^3^ This variability underscores the limitations of a ‘one-size-fits-all’ approach and highlights the need for personalized strategies to optimize the efficacy and safety of antiplatelet agents.^5^ To this extent, international consensus documents recommend the selective use of platelet function (PFT) or genetic testing in specific clinical scenarios to guide antiplatelet therapy.^4,6^ Notably, a recent analysis of a large cohort of patients undergoing PFT with light transmission aggregometry (LTA) showed hyper-reactive or hypo-reactive platelet phenotypes, not only in patients with concomitant antiplatelet therapy, but also in controls (CTR) and healthy volunteers (HV).^7^

Several clinical factors, including advanced age, diabetes mellitus (DM), chronic kidney disease (CKD), and certain ethnicities, have been identified as contributors to variability in platelet reactivity and are well-recognized indicators of thrombotic risk.^5,8^ However, the influence of sex on platelet reactivity and its prognostic significance remains a subject of debate.^9,10^ Emerging evidence suggests sex-specific differences in platelet biology and responses to antiplatelet agents, yet no specific recommendations addressing these differences have been provided in international guidelines or consensus statements.^11^ Moreover, existing studies comparing platelet reactivity between men and women are often limited by small sample sizes and methodological inconsistencies, which are susceptible to intra- and interlaboratory variability.^12,13^

On this background, we used a large single-centre dataset of patients undergoing platelet reactivity assessment by the gold standard LTA with a standardized and consistent methodology to investigate potential sex differences in platelet reactivity in subjects with or without concomitant antiplatelet therapy.

## Materials and methods

This retrospective analysis involved 11 913 individuals who underwent PFT at the Policlinico Umberto I University Hospital, Unit of Advanced Diagnosis Platelet Disorders (Rome, Italy) between 1 January 2004, and 31 December 2022. The complete database, as well as the detailed description of blood sampling and laboratory assessment procedures has been previously reported.^7^ In brief, LTA using a standardized methodology were assessed at 4 min and reported as percentage of platelet aggregation (PA%), using the following platelet agonists: (i) adenosine diphosphate (ADP) at the concentration of 2μM; collagen at the concentration of 2 μg/mL; epinephrine at the concentrations of 10 μM, arachidonic acid (AA) at the concentration of 0.5 and 0.75 μM.^7^

The population was categorized into five groups: (i) HV, including individuals without cardiovascular (CV) risk factors or concomitant antiplatelet therapy; (ii) CTR, including individuals with at least one CV risk factor (hypertension, DM, hypercholesterolaemia, previous thrombotic event) but not treated with antiplatelet therapy; (iii) patients on low-dose aspirin 75–150 mg/day (ASA); (iv) patients on clopidogrel 75 mg/day (CLOP); (v) patients on DAPT treated with a combination of ASA (75–150 mg/day) and CLOP (75 mg/day). Antiplatelet therapy, for either primary or secondary prevention, was prescribed at the treating physician’s discretion. In this study, such categories were divided into females (F) and males (M).

Exclusion criteria have been reported in detail in a previous study.^7^ Briefly, all patients presenting platelet disorders, abnormal bleeding, and every subject affected by diseases or taking therapies influencing PFT and those taking different dosages of antiplatelet therapies nor compliant.

### Study endpoints

The primary endpoint of the study was the difference in PA% measured 4-minute after exposure to the various platelet agonists (ADP, collagen, epinephrine, and AA) between females and males within each of the five groups of patients (HV, CTR, ASA, CLOP, and DAPT).

### Statistical analysis

Data were reported as median and interquartile ranges (IQRs) of the PA%. Patient categorical variables are represented as frequencies and percentages. Comparisons between categorical variables were conducted using two-tailed Fisher’s exact test or Pearson’s *χ*² test. Wilcoxon-Mann–Whitney *U* test was used for comparing continuous variables not normally distributed, as assessed by the Kolmogorov–Smirnov test. To mitigate selection bias across all groups (except HV), the Inverse Probability of Treatment Weighting (IPTW) method with Covariate Balancing Propensity Score (CBPS) was employed.^14^ In this approach, each patient is assigned a weight based on their propensity score to create a pseudo-population with balanced baseline characteristics. Specifically, CBPS optimizes both propensity score estimation and covariate balance between treatment groups. Following weighting, covariates were well balanced between treated and untreated groups. Importantly, in this weighted pseudo-population generated by IPTW, summary statistics no longer reflect raw patient counts but instead represent the sum of the weights assigned to individuals. Each subject contributes proportionally to their weight, and tables showing descriptive statistics are therefore interpreted in terms of weighted totals. Age, hypertension, hypercholesterolaemia, smoking, diabetes, CAD/myocardial infarction, peripheral artery disease, previous stroke/transient ischaemic attack (TIA), retinal thrombosis, carotid obstruction, beta-blocking agents, calcium channel blocker, angiotensin-converting enzyme-I, angiotensin receptor blockers/angiotensin 2, nitrates, statin, antidiabetic drugs, diuretic, omega-3 fatty acids were used for weights estimation. To address missing data on age, K-nearest neighbours imputation was applied. The absolute standardized mean differences (ASMD) were reported in order to assess balancing across groups; variables with ASMD < 0.1 were considered as balanced; weighted means (SD) and percentages were reported after IPTW, weighted medians and IQR for response to ADP (2 µM), collagen (2 µg/mL), epinephrine (10 µM), AA (0.5 mM), and AA (0.75 mM) were reported. A *P*-value lower than 0.05 was deemed statistically significant. All analyses were conducted using GraphPad Prism 9, version 9.1.0 and R 4.4.0 (R Foundation for Statistical Computing, Vienna, Austria). A pre-specified secondary analysis according to age (<50 years vs. >50 years) was performed within each of the five groups of patients.

## Results

A total of 5687 patients met study entry criteria: 428 HV (*F* = 273; *M* = 155), 1055 CTR (*F* = 725; *M* = 330); 3289 patients undergoing ASA (*F* = 2058; *M* = 1231), 430 patients undergoing clopidogrel (*F* = 272; *M* = 158), and 485 patients undergoing DAPT (*F* = 166; *M* = 319). Baseline characteristics and concomitant medications before and after IPTW are reported in Supplementary material online, *Table S1* for the HV group and in *Table 1* for the CTR, ASA, CLOP, and DAPT groups. No significant differences were observed between males and females in the HV group. However, in other groups, baseline characteristics such as hypertension, smoking, previous myocardial infarction, and prior ischaemic stroke or TIA exhibited some imbalances, which were adjusted after IPTW.

**Table 1:** Baseline characteristics and concomitant medications according to sex before and after IPTW in controls (CTR group), patients on aspirin therapy (ASA group), patients on clopidogrel therapy (CLOP group), and patients on aspirin plus clopidogrel therapy (DAPT group)

CTR							
	Before IPTW				After IPTW		
	F	M	*P*	ASMD	F	M	ASMD
n/sum of weights (IPTW)	725	330			1058.00	1058.00	
Age (mean ± SD, years)	63.98 (12.92)	63.38 (14.81)	0.529	0.043	63.96 (12.58)	63.96 (14.48)	0.028
**Clinical conditions and risk factors**							
Hypertension, *n* (%)	422 (58.2)	227 (68.8)	0.001	0.221	654.6 (61.9)	654.6 (61.9)	<0.001
Hypercholesterolaemia, *n* (%)	276 (38.1)	110 (33.3)	0.148	0.099	388.5 (36.7)	388.5 (36.7)	<0.001
Smoking, *n* (%)	152 (21.0)	76 (23.0)	0.468	0.050	225.0 (21.3)	225.0 (21.3)	<0.001
Diabetes, *n* (%)	67 (9.2)	41 (12.4)	0.125	0.103	105.5 (10.0)	105.5 (10.0)	<0.001
CAD/MI, *n* (%)	5 (0.7)	5 (1.5)	0.301	0.079	10.8 (1.0)	10.8 (1.0)	<0.001
PAD, *n* (%)	9 (1.2)	1 (0.3)	0.186	0.107	9.4 (0.9)	9.4 (0.9)	<0.001
Previous stroke/TIA, *n* (%)	197 (27.2)	99 (30.0)	0.375	0.063	294.2 (27.8)	294.2 (27.8)	<0.001
Retinal thrombosis, *n* (%)	10 (1.4)	3 (0.9)	0.765	0.044	12.0 (1.1)	12.0 (1.1)	<0.001
Carotid obstruction, *n* (%)	47 (6.5)	17 (5.2)	0.487	0.057	62.2 (5.9)	62.2 (5.9)	<0.001
**Medications**							
Beta-blocking agents, *n* (%)	125 (17.2)	45 (13.6)	0.149	0.100	169.3 (16.0)	169.3 (16.0)	<0.001
Calcium channel blocker, *n* (%)	117 (16.1)	60 (18.2)	0.424	0.054	171.9 (16.2)	171.9 (16.2)	<0.001
ACE-I, *n* (%)	119 (16.4)	80 (24.2)	0.003	0.195	208.3 (19.7)	208.3 (19.7)	<0.001
ARBs/angiotensin 2, *n* (%)	136 (18.8)	84 (25.5)	0.014	0.162	219.9 (20.8)	219.9 (20.8)	<0.001
Nitrates, *n* (%)	9 (1.2)	6 (1.8)	0.575	0.047	14.8 (1.4)	14.8 (1.4)	<0.001
Statin, *n* (%)	157 (21.7)	55 (16.7)	0.068	0.127	207.8 (19.6)	207.8 (19.6)	<0.001
Antidiabetic drugs, *n* (%)	61 (8.4)	38 (11.5)	0.112	0.104	97.5 (9.2)	97.5 (9.2)	<0.001
Diuretic, *n* (%)	43 (5.9)	15 (4.5)	0.387	0.062	55.3 (5.2)	55.3 (5.2)	<0.001
Omega-3, *n* (%)	0.03 (0.16)	0.05 (0.22)	0.050	0.123	0.04 (0.18)	0.04 (0.18)	<0.001

ASA							
	Before IPTW				After IPTW		
	F	M	*P*	ASMD	F	M	ASMD
n/sum of weights (IPTW)	2058	1231			3288.50	3288.50	
Age (mean ± SD, years)	67.95 (12.03)	69.16 (11.62)	0.012	0.102	68.31 (10.91)	68.31 (11.12)	0.002
**Clinical conditions and risk factors**							
Hypertension, *n* (%)	1179 (57.3)	759 (61.7)	0.014	0.089	1930.2 (58.7)	1930.2 (58.7)	<0.001
Hypercholesterolaemia, *n* (%)	734 (35.7)	502 (40.8)	0.004	0.105	1234.4 (37.5)	1234.4 (37.5)	<0.001
Smoking, *n* (%)	229 (11.1)	232 (18.8)	<0.001	0.218	453.1 (13.8)	453.1 (13.8)	<0.001
Diabetes, *n* (%)	175 (8.5)	139 (11.3)	0.010	0.093	313.0 (9.5)	313.0 (9.5)	<0.001
CAD/MI, *n* (%)	30 (1.5)	27 (2.2)	0.129	0.055	55.1 (1.7)	55.1 (1.7)	<0.001
PAD, *n* (%)	21 (1.0)	12 (1.0)	1.000	0.005	32.9 (1.0)	32.9 (1.0)	<0.001
Previous stroke/TIA, *n* (%)	534 (25.9)	327 (26.6)	0.712	0.014	862.8 (26.2)	862.8 (26.2)	<0.001
Retinal thrombosis, *n* (%)	43 (2.1)	20 (1.6)	0.430	0.034	66.1 (2.0)	66.1 (2.0)	<0.001
Carotid obstruction, *n* (%)	117 (5.7)	125 (10.2)	<0.001	0.166	249.4 (7.6)	249.4 (7.6)	<0.001
**Medications**							
Beta-blocking agents, *n* (%)	328 (15.9)	229 (18.6)	0.049	0.071	553.8 (16.8)	553.8 (16.8)	<0.001
Calcium channel blocker, *n* (%)	380 (18.5)	206 (16.7)	0.221	0.045	600.6 (18.3)	600.6 (18.3)	<0.001
ACE-I, *n* (%)	397 (19.3)	343 (27.9)	<0.001	0.203	743.2 (22.6)	743.2 (22.6)	<0.001
ARBs/angiotensin 2, *n* (%)	459 (22.3)	250 (20.3)	0.189	0.049	702.0 (21.3)	702.0 (21.3)	<0.001
Nitrates, *n* (%)	50 (2.4)	69 (5.6)	<0.001	0.162	114.2 (3.5)	114.2 (3.5)	<0.001
Statin, *n* (%)	560 (27.2)	382 (31.0)	0.021	0.084	936.6 (28.5)	936.6 (28.5)	<0.001
Antidiabetic drugs, *n* (%)	154 (7.5)	108 (8.8)	0.206	0.047	262.6 (8.0)	262.6 (8.0)	<0.001
Diuretic, *n* (%)	156 (7.6)	90 (7.3)	0.837	0.010	247.6 (7.5)	247.6 (7.5)	<0.001
Omega-3, *n* (%)	0.03 (0.18)	0.07 (0.25)	<0.001	0.161	0.05 (0.21)	0.05 (0.21)	<0.001

CLOP							
	Before IPTW				After IPTW		
	F	M	*P*	ASMD	F	M	ASMD
n/sum of weights (IPTW)	272	158			431.8	433.8	
Age (mean ± SD, y)	71.32 (9.28)	73.46 (9.90)	0.034	0.223	72.20 (8.72)	72.12 (10.16)	0.009
**Clinical conditions and risk factors**							
Hypertension, *n* (%)	180 (66.2)	106 (67.1)	0.916	0.019	288.5 (66.8)	290.5 (67.0)	0.003
Hypercholesterolaemia, *n* (%)	147 (54.0)	53 (33.5)	<0.001	0.422	203.7 (47.2)	205.7 (47.4)	0.005
Smoking, *n* (%)	36 (13.2)	50 (31.6)	<0.001	0.452	90.5 (20.9)	90.5 (20.9)	0.002
Diabetes, *n* (%)	30 (11.0)	25 (15.8)	0.178	0.141	50.1 (11.6)	52.1 (12.0)	0.012
CAD/MI, *n* (%)	9 (3.3)	4 (2.5)	0.776	0.046	13.5 (3.1)	13.5 (3.1)	0.001
PAD, *n* (%)	0 (0.0)	2 (1.3)	0.134	0.160	0.0 (0.0)	2.0 (0.5)	0.058
Previous stroke/TIA, *n* (%)	157 (57.7)	67 (42.4)	0.003	0.310	225.8 (52.3)	225.8 (52.1)	0.005
Retinal thrombosis, *n* (%)	7 (2.6)	1 (0.6)	0.268	0.155	8.9 (2.1)	8.9 (2.1)	0.001
Carotid obstruction, *n* (%)	26 (9.6)	10 (6.3)	0.282	0.120	37.3 (8.6)	37.3 (8.6)	0.002
**Medications**							
Beta-blocking agents, *n* (%)	62 (22.8)	28 (17.7)	0.222	0.126	91.7 (21.2)	91.7 (21.1)	0.003
Calcium channel blocker, *n* (%)	50 (18.4)	34 (21.5)	0.450	0.079	81.8 (18.9)	81.8 (18.9)	0.002
ACE-I, *n* (%)	42 (15.4)	29 (18.4)	0.501	0.078	69.1 (16.0)	69.1 (15.9)	0.002
ARBs/angiotensin 2, *n* (%)	77 (28.3)	49 (31.0)	0.583	0.059	128.9 (29.9)	128.9 (29.7)	0.003
Nitrates, *n* (%)	12 (4.4)	5 (3.2)	0.615	0.065	19.7 (4.6)	19.7 (4.5)	0.001
Statin, *n* (%)	116 (42.6)	44 (27.8)	0.003	0.314	165.9 (38.4)	167.9 (38.7)	0.006
Antidiabetic drugs, *n* (%)	29 (10.7)	24 (15.2)	0.174	0.135	48.9 (11.3)	50.9 (11.7)	0.012
Diuretic, *n* (%)	28 (10.3)	4 (2.5)	0.002	0.321	29.8 (6.9)	29.8 (6.9)	0.001
Omega-3, *n* (%)	0.08 (0.27)	0.10 (0.30)	0.392	0.084	0.09 (0.29)	0.09 (0.29)	0.001

DAPT							
	Before IPTW				After IPTW		
	F	M	*P*	ASMD	F	M	ASMD
*n*/sum of weights (IPTW)	165	319			501.1	503.1	
Age (mean ± SD, years)	69.94 (11.73)	61.97 (13.32)	<0.001	0.635	62.87 (13.54)	62.91 (10.72)	0.004
**Clinical conditions and risk factors**							
Hypertension, *n* (%)	88 (53.3)	174 (54.5)	0.848	0.024	274.2 (54.7)	275.2 (54.7)	<0.001
Hypercholesterolaemia, *n* (%)	59 (35.8)	165 (51.7)	0.001	0.326	234.5 (46.8)	235.5 (46.8)	<0.001
Smoking, *n* (%)	10 (6.1)	20 (6.3)	1.000	0.009	38.8 (7.7)	39.8 (7.9)	0.007
Diabetes, *n* (%)	14 (8.5)	23 (7.2)	0.594	0.047	51.7 (10.3)	52.7 (10.5)	0.006
CAD/MI, *n* (%)	43 (26.1)	23 (7.2)	<0.001	0.523	66.4 (13.3)	66.4 (13.2)	0.002
PAD, *n* (%)	0 (0.0)	1 (0.3)	1.000	0.079	0.0 (0.0)	1.0 (0.2)	0.050
Previous stroke/TIA, *n* (%)	29 (17.6)	18 (5.6)	<0.001	0.379	44.8 (8.9)	44.8 (8.9)	0.001
Retinal thrombosis, *n* (%)	0 (0.0)	1 (0.3)	1.000	0.079	0.0 (0.0)	1.0 (0.2)	0.050
Carotid obstruction, *n* (%)	7 (4.2)	12 (3.8)	0.808	0.025	32.0 (6.4)	33.0 (6.6)	0.009
**Medications**							
Beta-blocking agents, *n* (%)	25 (15.2)	105 (32.9)	<0.001	0.425	128.0 (25.5)	129.0 (25.6)	0.002
Calcium channel blocker, *n* (%)	33 (20.0)	18 (5.6)	<0.001	0.440	60.4 (12.1)	61.4 (12.2)	0.005
ACE-I, *n* (%)	27 (16.4)	84 (26.3)	0.016	0.245	125.8 (25.1)	125.8 (25.0)	0.002
ARBs/angiotensin 2, *n* (%)	33 (20.0)	30 (9.4)	0.002	0.303	47.9 (9.6)	47.9 (9.5)	0.001
Nitrates, *n* (%)	11 (6.7)	31 (9.7)	0.308	0.111	39.1 (7.8)	39.1 (7.8)	0.001
Statin, *n* (%)	48 (29.1)	140 (43.9)	0.002	0.311	205.1 (40.9)	206.1 (41.0)	0.001
Antidiabetic drugs, *n* (%)	10 (6.1)	14 (4.4)	0.508	0.075	38.5 (7.7)	39.5 (7.8)	0.008
Diuretic, *n* (%)	12 (7.3)	26 (8.2)	0.859	0.033	42.4 (8.5)	42.4 (8.4)	0.001
Omega-3, *n* (%)	0.09 (0.29)	0.18 (0.39)	0.008	0.267	0.13 (0.34)	0.13 (0.34)	0.002

ACE, angiotensin-converting enzyme; ARBs, angiotensin receptor blockers; CAD, coronary artery disease; F, females; M, males; MI, myocardial infarction; PAD, peripheral artery disease; SD, standard deviation; TIA, transient ischaemic attack; IPTW, inverse probability of treatment weighting; ASMD, absolute standardized mean differences.

### Healthy volunteers

**Figure 1:**
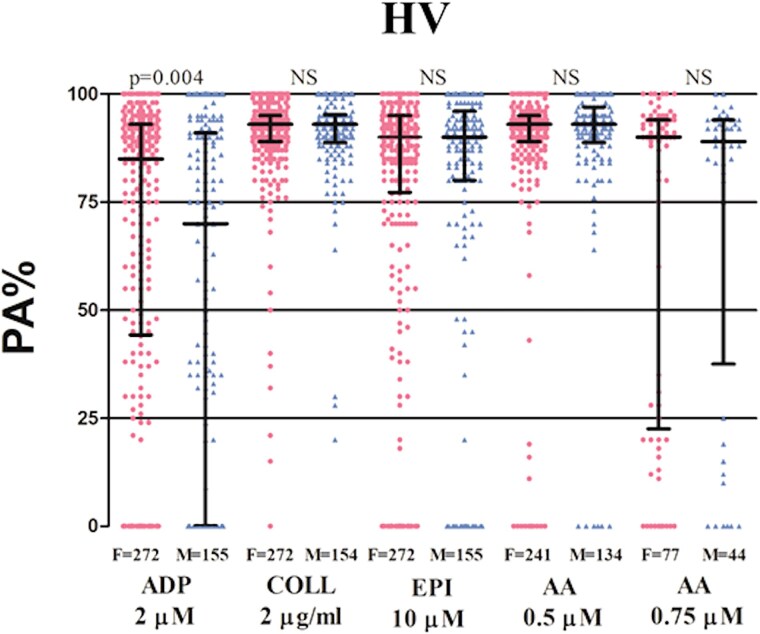
Scatter dot plot of platelet reactivity assessed by light transmission aggregometry following adenosine diphosphate 2 µM, collagen 2 µg/mL, epinephrine 10 µM, and arachidonic acid 0.5 or 0.75 µM in healthy volunteers stratified by sex. Lines represent median with IQR range. N refers to the number of patients analysed. HV, healthy volunteers; IQR, interquartile range; F, females; M, males; LTA, light transmission aggregometry; ADP, adenosine diphosphate; COLL, collagen; EPI, epinephrine; AA, arachidonic acid; NS, non-significant; PA, platelet aggregation.

Median PA% was significantly increased in HV in response to ADP (85 [45–93] vs. 70 [0–91]; *P* = 0.004) in females compared to males. There were no differences between females and males in response to collagen (93 [89–95] vs. 93 [89–95]; *P* = 0.903), epinephrine (90 [78–95] vs. 90 [80–95]; *P* = 0.446), and AA (93 [89–95] vs. 93 [89–97]; *P* = 0.442) (*Figure 1* and see Supplementary material online, *Table S2*).

At the analysis according to age, females <50 years showed lower PA% in response to epinephrine (88 [75–93] vs. 90 [86–95]; *P* = 0.005), compared with females >50 years (*Table 2*). There were no differences in PA% between females <50 or >50 years old in response to other stimuli (*Table 2*). There were no differences in PA% according to age in response to any stimuli in males (*Table 2*).

**Table 2:** Summary of statistical parameters for healthy volunteers based on age (<50 years old vs. > 50 years old.) in response to ADP (2 µM), collagen (2 µg/mL), epinephrine (10 µM), and arachidonic acid (0.5 mM)

Agonist (concentration) *N*°	Median (IQR)		*P*-value
	female > 50 y.o.	female < 50 y.o.	
ADP (2 µM) 81/146	88 (49–93)	82 (40–92)	0.190
Epinephrine (10 µM) 81/146	90 (86–95)	88 (75–93)	**0.005**
Collagen (2 µg/mL) 81/146	94 (90–96)	93 (89–95)	0.355
AA (0.5 mM) 76/128	94 (90–95)	93 (88–95)	0.097
	**males > 50 y.o.**	**male < 50 y.o.**	
ADP (2 µM) 49/74	70 (0–91)	75 (20–91)	0.632
Epinephrine (10 µM) 49/74	89 (55–93)	90 (80–94)	0.160
Collagen (2 µg/mL) 48/74	92 (88–95)	93 (90–95)	0.107
AA (0.5 mM) 44/59	92 (88–95)	94 (90–99)	0.348

Values are reported as platelet aggregation percentage at 4 min through mean ± standard deviation (SD), median (25–75% interquartile ranges).

ADP, adenosine diphosphate; IQR, interquartile range. y.o., years old.

### Controls

**Figure 2:**
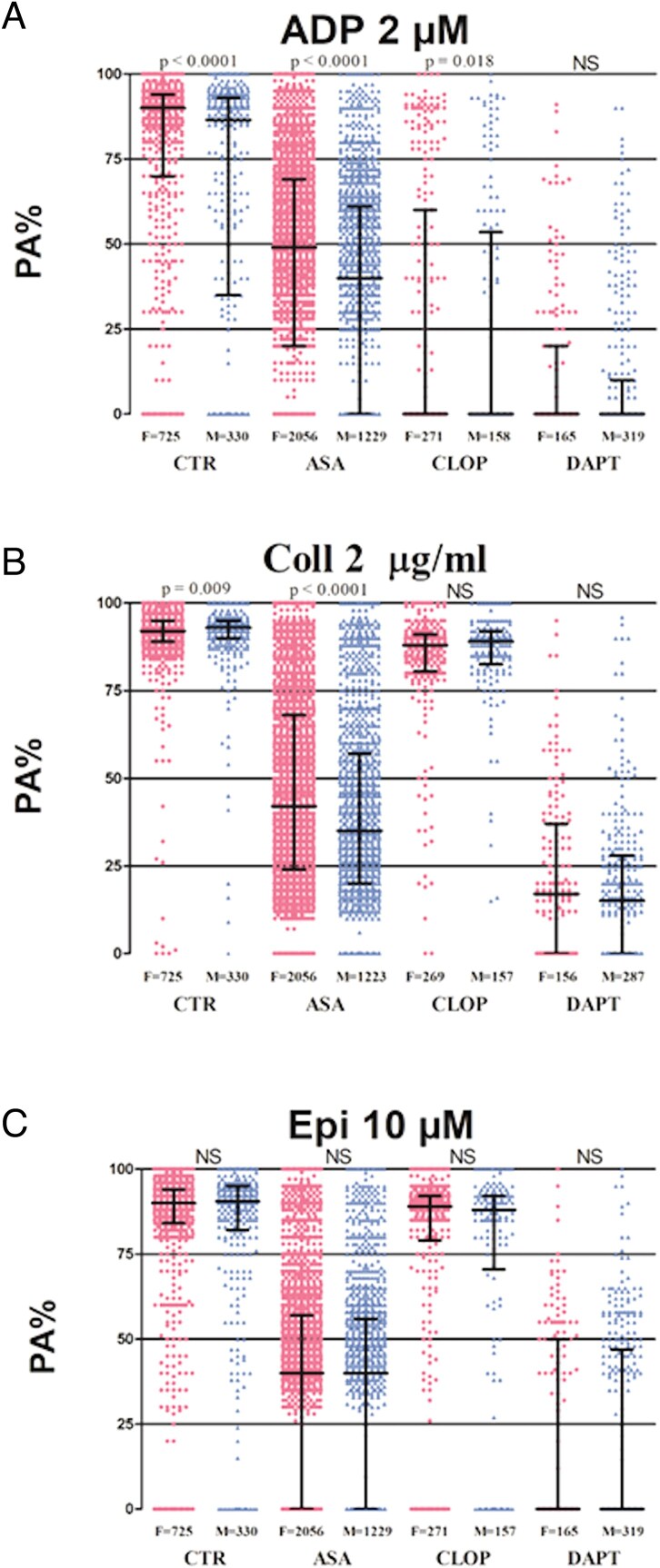
Scatter dot plot of platelet reactivity assessed by light transmission aggregometry following ADP 2 µM (*A*), collagen 2 µg/mL (*B*), and epinephrine 10 µM (*C*) in the control group, aspirin group, clopidogrel group, dual antiplatelet therapy group populations stratified by sex. Lines represent median with interquartile range range. *N* refers to the number of patients analysed. CTR, control group; ASA, aspirin group; CLOP, clopidogrel group; DAPT, dual antiplatelet therapy group; IQR, interquartile range; F, females; M, males; LTA, light transmission aggregometry; ADP, adenosine diphosphate; COLL, collagen; EPI, epinephrine; AA, arachidonic acid; NS, non-significant; PA, platelet aggregation.

**Figure 3:**
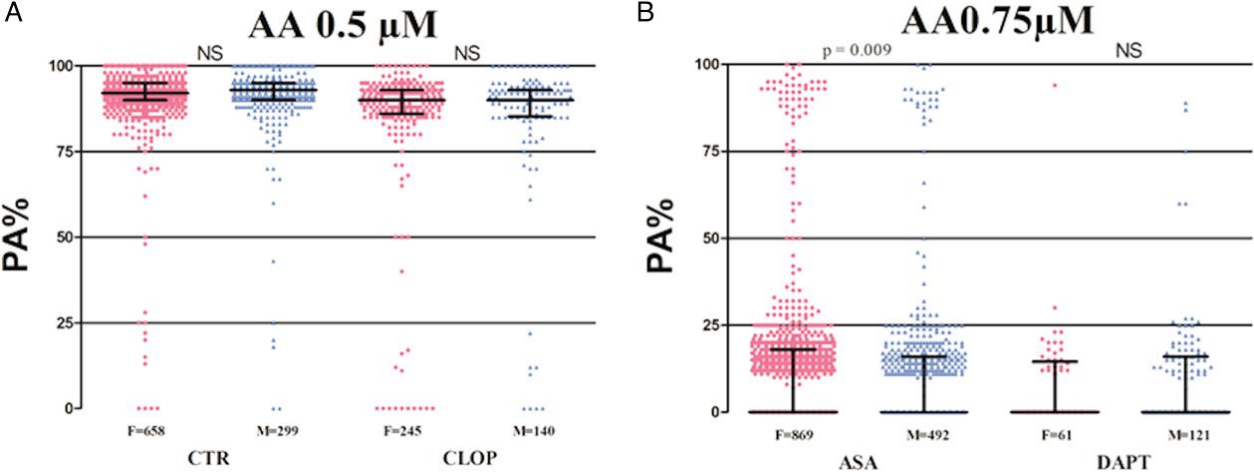
Scatter dot plot of platelet reactivity assessed by light transmission aggregometry following AA 0.5 mM in the control group and in the clopidogrel group populations (*A*) or AA 0.75 mM for the aspirin group and the dual antiplatelet therapy group populations (*B*), stratified by sex. Lines represent median with interquartile range range. *N* refers to the number of patients analysed. Abbreviations: CTR, control group; ASA, aspirin group; CLOP, clopidogrel group; DAPT, dual antiplatelet therapy group; IQR, interquartile range; F, females; M, males; LTA, light transmission aggregometry; ADP, adenosine diphosphate; COLL, collagen; EPI, epinephrine; AA, arachidonic acid; NS, non-significant; PA, platelet aggregation.

Weighted median PA% was significantly increased in CTR in response to ADP (90 [70–94] vs. 86 [37–93]; *P* < 0.0001) in females compared with males (*Figure 2A* and see Supplementary material online, *Table S3*). There was a statistically significant but numerically minimal difference in median PA% between females and males in response to collagen (92 [89–95] vs. 93 [90–95]; *P* = 0.009) (*Figure 2B* and see Supplementary material online, *Table S3*). There were no differences in mean PA% between females and males in response to epinephrine (90 [84–94] vs. 90 [83–95]; *P* = 0.732) (*Figure 2C* and see Supplementary material online, *Table S3*), and AA (92 [90–95] vs. 93 [90–95]; *P* = 0.157) (*Figure 3A* and see Supplementary material online, *Table S3*).

### Aspirin-treated patients

Weighted median PA% in ASA patients was significantly increased in females compared to males in response to ADP (49 [20–68] vs. 40 [0–60]; *P* < 0.0001) (*Figure 2A* and see Supplementary material online, *Table S3*) and collagen (42 [24–68] vs. 35 [20–57]; *P* < 0.001) (*Figure 2B* and see Supplementary material online, *Table S3*). There was a statistically significant but numerically minimal difference in median PA% between females and males in response to AA (15 [0–25] vs. 14 [0–22]; *P* = 0.009) (*Figure 3B* and see Supplementary material online, *Table S3*). There were no differences in median PA% between females and males in response to epinephrine (40 [0–56] vs. 39 [0–55]; *P* = 0.18) (*Figure 2C* and see Supplementary material online, *Table S3*).

### Clopidogrel-treated patients

Weighted median PA% in CLOP patients was significantly increased in females compared to males in response to ADP (0 [0–65] vs. 0 [0–40; *P* = 0.018) (*Figure 2A* and see Supplementary material online, *Table S3*), but not to collagen (88 [82–90] vs. 88 [82–91]; *P* = 0.568) (*Figure 2B* and see Supplementary material online, *Table S3*), epinephrine (90 [80–93] vs. 88 [72–92]; *P* = 0.134) (*Figure 2C* and see Supplementary material online, *Table S3*), and AA (90 [86–93] vs. 90 [85–93]; *P* = 0.476) (*Figure 3A* and see Supplementary material online, *Table S3*).

### Dual antiplatelet therapy-treated patients

There were no differences in weighted median PA% between females and males in DAPT patients in response to ADP (0 [0–30] vs. 0 [0–10]; *P* = 0.169) (*Figure 2A* an see Supplementary material online, *Table S3*), collagen (18 [0–40] vs. 17 [0–28]; *P* = 0.242) (*Figure 2B* and see Supplementary material online, *Table S3*), epinephrine (0 [0–55] vs. 0 [0–46]; *P* = 0.556) (*Figure 2C* and see Supplementary material online, *Table S3*), and AA (15 [12–50] vs. 0 [0–20]; *P* = 0.091) (*Figure 3B* and see Supplementary material online, *Table S3*).

## Discussion

To the best of our knowledge, this is the largest study to evaluate sex differences in platelet reactivity using the gold standard LTA with multiple agonists, following a standardized and consistent methodology, both in the presence and absence of antiplatelet therapy. The main findings of the study can be summarized as follows: (i) there was significant interindividual variability in platelet reactivity in response to all agonists in both females and males; (ii) among CTR and HV, females showed increased platelet reactivity as assessed by LTA following ADP stimulus, compared to males; (iii) among patients treated with clopidogrel, females showed higher platelet reactivity assessed by LTA following ADP, compared with males; (iv) among patients treated with aspirin, females showed higher platelet reactivity assessed by LTA following ADP, collagen, and AA stimulus, compared with males; (v) platelet reactivity in response to all stimuli was similar between females and males in the DAPT group; (vi) females in the HV group older than 50 exhibited increased platelet reactivity in response to epinephrine compared to those younger than 50.

Platelet inhibition is the cornerstone for the treatment and prevention of thrombotic complications in ASCVD.^1^ In this context, two main classes of oral antiplatelet drugs are currently recommended: aspirin and P2Y_12_ receptor antagonists (i.e. clopidogrel, prasugrel, or ticagrelor).^6^ Aspirin exerts its antiplatelet effect by irreversibly inhibiting Cyclooxygenase-1 (COX-1) in platelets, leading to a reduction in thromboxane A2 (TXA_2_) production, a potent promoter of platelet activation.^15^ P2Y_12_ inhibitors block ADP-mediated platelet activation.^15^ Activation of the P2Y_12_ receptor triggers a signalling cascade essential for platelet activation and fibrinogen binding, ultimately facilitating PA and thrombus formation.

A substantial body of evidence has demonstrated that oral antiplatelet agents, particularly the P2Y_12_ inhibitor clopidogrel, exhibit significant variability in individual response.^3,16,17^ This variability can lead to either inadequate (HPR) or excessive platelet inhibition (LPR), both of which have important clinical implications.^3,16,17^ Notably, growing evidence suggests that a significant proportion of individuals not receiving antiplatelet therapy exhibit hyper- or hypo-reactive platelet phenotypes, which may influence their clinical history as well as the safety and efficacy of antiplatelet treatment.^7,18^ Several clinical factors, including advanced age, DM, CKD, and certain ethnicities, have been identified as contributors to heightened platelet reactivity and are well-recognized indicators of thrombotic risk.^5,8^ However, the influence of sex on platelet reactivity and its prognostic significance remains an area of ongoing investigation.^10^ While international guidelines recommend specific antiplatelet strategies for certain high-risk populations, no tailored recommendations exist according to sex.^1,4^ These recommendations stem from limited clinical evidence, largely due to the under-representation of females in RCTs, as well as the conflicting results of pharmacodynamic (PD) studies.^13,19–24^ However, current PD studies are limited in sample size and are often hindered by methodological inconsistencies in platelet reactivity assessment, making comparisons challenging.^13,19–24^

Recently, there has been significant interest in precision medicine, aimed at optimizing the risk-benefit profile of antiplatelet therapies, given the inherent risk of bleeding associated with these treatments.^5^ To this end, integrating ischaemic and bleeding risk scoring systems with tools allowing for the appraisal or estimation of the response to antiplatelet therapy (i.e. PFT or genetic testing) has been found to improve outcomes and is recommended by a recent international consensus document.^4^ With regards to PFT, several bedside or laboratory tests have been proposed for guiding antiplatelet selection in patients undergoing percutaneous coronary interventions.^25^ Among these, LTA is a laboratory test considered the historical gold standard, but has limitations, including complex sample preparation requiring expert personnel and the inter- and intra-laboratory variability.^4^ Platelet aggregation assessment by LTA may be induced by several agonists, each reflecting specific pathways involved in thrombus formation. Light transmission aggregometry induced by ADP serves as a marker of P2Y_12_ receptor activity, making it a valuable test for assessing individual responses to P2Y_12_ inhibitors.^7^ Conversely, LTA stimulated by collagen, AA, and epinephrine allow to unravel activation of the TXA_2_ pathway and more suitable for monitoring aspirin therapy, although AA has been shown to be less reliable for this purpose.^7^

In our analysis, we confirmed that the large interindividual variability in platelet reactivity observed in both patients receiving and not receiving antiplatelet therapy was similar between females and males.^7^ Furthermore, we observed that HV and CTR females exhibited higher platelet reactivity in response to ADP compared to males. This difference persisted in patients receiving clopidogrel but was abolished in those undergoing DAPT. Moreover, females treated with ASA showed higher platelet reactivity in response to collagen compared to males, suggesting a sex difference in aspirin responsiveness. These findings align with and expand on those reported by other groups.^24,26^ Compared to previous studies, our work benefits from a larger sample size, standardized methodology using gold standard LTA, and inclusion of both patients on antiplatelet therapy and HV or CTR. Additionally, we employed a comprehensive panel of agonists (ADP, collagen, epinephrine, and AA) to assess PFT across multiple activation pathways. The observation that females exhibit baseline hyperreactivity of the P2Y_12_ pathway but a reduced response to aspirin compared to males may have important clinical implications, suggesting that P2Y_12_ inhibitors may be particularly beneficial in women. These PD findings may have important clinical implications, and are consistent with evidence from an individual participant data meta-analysis including 24 096 patients from six RCTs, which indicates that P2Y_12_ inhibitor monotherapy is especially effective in women compared to men.^27^ Additionally, the observation that females treated with clopidogrel monotherapy exhibit heightened platelet reactivity in response to ADP suggests that women may derive greater benefit than men from a guided selection of P2Y_12_ inhibitors using PFT or genetic testing or from the use of more potent P2Y_12_ inhibitor, such as prasugrel or ticagrelor. While these experimental findings hold the potential to significantly influence clinical practice, they remain hypothesis-generating and support the need for rigorous clinical trials that could ultimately influence guideline recommendations and clinical decision-making.

Finally, in a secondary analysis stratified by age, we found that, among HV, females—but not males—exhibited greater platelet reactivity with advancing age. This finding aligns with previous studies and supports clinical observations that CV risk increases significantly in women after the postmenopausal transition.^28^ This increase is likely influenced by factors such as hormonal changes, genetic variations affecting COX-1 inhibition, and other sex-specific differences that may contribute to variability in response to antiplatelet therapy.^29^

Overall, this large PD study, which assessed platelet reactivity using the gold standard LTA with a standardized methodology, highlights significant sex differences in platelet reactivity both with and without antiplatelet therapy. Sex-related disparities in clot formation have also been highlighted using other point-of-care assays, including thromboelastography, a global clotting assay, further supporting the existence of biologically driven differences in thrombus formation between males and females.^30,31^ These findings provide a rationale for the growing clinical evidence supporting sex-specific responses to different antiplatelet agents and the potential to for considering sex in a precision medicine approach. For this purpose, PFT and genetics may offer advantages in modern setting.^4,25^

### Limitations

As an observational, PD, study, this analysis has several limitations, including the inability to establish causal relationships (only associations), potential confounding due to uncontrolled variables, and the possibility of recall bias affecting the results. Additionally, we were unable to account for treatment duration among patients undergoing SAPT or DAPT. However, patients who did not adhere to the prescribed antiplatelet therapy in the seven days preceding blood withdrawal were excluded. Differences in antiplatelet therapy duration beyond seven days are unlikely to significantly affect the comparison of platelet reactivity between females and males.

Our analysis included only clopidogrel as the P2Y_12_ inhibitor. Given the significant PD and clinical differences between clopidogrel and more potent and predictable P2Y_12_ inhibitors, our findings apply specifically to clopidogrel and cannot be generalized to the entire class of P2Y_12_ inhibitors. Further research is needed to explore sex-related differences in platelet reactivity with other P2Y_12_ inhibitors, such as prasugrel or ticagrelor.^32^ Lastly, we did not stratify results by genetic testing (*CYP2C19*) in the clopidogrel and DAPT groups.

## Conclusions

This large PD study highlights significant sex differences in platelet reactivity, both in the presence and absence of antiplatelet therapy. The higher baseline platelet reactivity linked to the P2Y_12_ receptor pathway and the lower response to aspirin in females compared to males suggest that P2Y_12_ inhibitors may be the preferred antiplatelet therapy for women. These observations may explain findings from clinical studies suggesting that P2Y_12_ inhibitor monotherapy might be especially effective in women compared to men.

## Supplementary Material

pvaf034_Supplementary_Data

## Data Availability

The data underlying this article will be shared on reasonable request to the corresponding author.
