# Supplementary material for: Sex-related variations in platelet reactivity in presence or absence of antiplatelet therapy
Source: Eur Heart J Cardiovasc Pharmacother. 2025 May 14;11(6):509–17. doi: 10.1093/ehjcvp/pvaf034 (PMC12450592; doi:10.1093/ehjcvp/pvaf034)
Supplement: pvaf034_Supplementary_Data [file pvaf034_supplementary_data.docx]

**SUPPLEMENTAL METHODS**

**Collection and Sample preparation**

Blood withdrawal was collected using vacuette vacutainer 10 ml anticoagulated with 3.8% sodium citrate (Greiner Bio-One), with a 1:10 ratio (v:v), and maintained at room temperature (RT), and in less than one hour platelet rich plasma (PRP) was prepared.

Citrated blood was centrifuged at 200xg for 15 minutes at RT for PRP preparation. After separation PRP tube were further centrifuge for 10 minutes at 2000xg at RT, for Platelet Poor Plasma (PPP).

Platelet aggregation has been evaluated in PRP in a four channel aggregometer (AggRam, Helena Laboratories, Beaumont, Texas, United States) according to the Born’s method(1).

Instrument calibration was performed according to the manufactory indications.

**Platelet Aggregation**

ADP (2 μM), Collagen (2 μg/ml), epinephrine (10 μM) and arachidonic acid (0.5-0.75 mM) were used as agonists. We report only the data of concentrations selected for each agonist as there was the minimal concentration that allow to obtain a higher aggregation percentage in more than 70% of HV.

The PA parameter that we used is the platelet aggregation percentage (PA%) at 4 minutes. This parameter has the advantage to avoid errors due to maximum primary aggregations where it is not particularly clear whether the response is primary or secondary.

**SUPPLEMENTAL RESULTS**

**Table S1.** Baseline characteristics and concomitant medications before and after IPTW are reported for the healthy volunteer group. Abbreviations: ACE, angiotensin-converting enzyme; ARBs, angiotensin receptor blockers; CAD, coronary artery disease; F, females; M, males; MI, myocardial infarction; PAD, peripheral artery disease; SD, standard deviation; TIA, transient ischemic attack; IPTW: inverse probability of treatment weighting; ASMD: absolute standardized mean differences.

| **HV** | | | | | | | |
| --- | --- | --- | --- | --- | --- | --- | --- |
|  | F | M | p | ASMD |  |  |  |
| n | 272 | 154 |  |  |  |  |  |
| Age (mean ± SD, y) | 42.31 (19.80) | 42.83 (22.37) | 0.826 | 0.024 |  |  |  |
| **Clinical conditions and risk factors** |  |  |  |  |  |  |  |
| Hypertension, n (%) | 0 (0.0) | 0 (0.0) | / | <0.001 |  |  |  |
| Hypercholesterolaemia, n (%) | 0 (0.0) | 0 (0.0) | / | <0.001 |  |  |  |
| Smoking, n (%) | 0 (0.0) | 0 (0.0) | / | <0.001 |  |  |  |
| Diabetes, n (%) | 0 (0.0) | 0 (0.0) | / | <0.001 |  |  |  |
| CAD/MI, n (%) | 0 (0.0) | 0 (0.0) | / | <0.001 |  |  |  |
| PAD, n (%) | 0 (0.0) | 0 (0.0) | / | <0.001 |  |  |  |
| Previous stroke/TIA, n (%) | 0 (0.0) | 0 (0.0) | / | <0.001 |  |  |  |
| Retinal thrombosis, n (%) | 0 (0.0) | 0 (0.0) | / | <0.001 |  |  |  |
| Carotid obstruction, n (%) | 0 (0.0) | 0 (0.0) | / | <0.001 |  |  |  |
| **Medications** |  |  |  |  |  |  |  |
| Beta-blocking agents, n (%) | 0 (0.0) | 0 (0.0) | / | <0.001 |  |  |  |
| Calcium channel blocker, n (%) | 0 (0.0) | 0 (0.0) | / | <0.001 |  |  |  |
| ACE-I, n (%) | 0 (0.0) | 0 (0.0) | / | <0.001 |  |  |  |
| ARBs/angiotensin 2, n (%) | 0 (0.0) | 0 (0.0) | / | <0.001 |  |  |  |
| Nitrates, n (%) | 0 (0.0) | 0 (0.0) | / | <0.001 |  |  |  |
| Statin, n (%) | 0 (0.0) | 0 (0.0) | / | <0.001 |  |  |  |
| Antidiabetic drugs, n (%) | 0 (0.0) | 0 (0.0) | / | <0.001 |  |  |  |
| Diuretic, n (%) | 0 (0.0) | 0 (0.0) | / | <0.001 |  |  |  |
| Omega-3, n (%) | 0.00 (0.00) | 0.00 (0.00) | / | <0.001 |  |  |  |

**Table S2.** Summary of statistical parameters for healthy volunteers in response to ADP (2 µM), collagen (2 µg/ml), epinephrine (10µM) and arachidonic acid (0.5mM). Values are reported as platelet aggregation percentage at 4 min through mean±standard deviation (SD), median (25-75% interquartile ranges). P-values test for differences between females (F) and males (M) in the same population. ADP, adenosine diphosphate; IQR, interquartile range.

| **Agonist (concentration) N F/M** | **Median (IQR)** | | **P-value** |
| --- | --- | --- | --- |
|  | Female | Male |  |
| ADP (2 µM)  272/155 | 85 (45-93) | 70 (0-91) | 0.004 |
| Collagen (2 µg/ml) 272/154 | 93 (89-95) | 93 (89-95) | 0.903 |
| Epinephrine (10 µM) 272/155 | 90 (78-95) | 90 (80-95) | 0.446 |
| AA (0.5 mM)  241/134 | 93 (89-95) | 93 (89-97) | 0.442 |

**Table S3.** Summary of statistical parameters for each studied population (except healthy volunteers which are presented in Table s) in response to ADP (2 µM), collagen (2 µg/ml), epinephrine (10µM) and arachidonic acid (0.5 for CTR and CLOP, and 0.75 mM for ASA and DAPT). Values are reported as platelet aggregation percentage at 4 min through mean±standard deviation (SD), median (25-75% interquartile ranges). P-values test for differences between females (F) and males (M) in the same population. ADP, adenosine diphosphate; ASA, aspirin; CLOP, clopidogrel; CTR,

control group; DAPT, double antiplatelet treatment; IQR, interquartile range.

| **Population (N F/M)** | **Weighted Median (IQR)** | | **P-value** |
| --- | --- | --- | --- |
|  | Female | Male |  |
|  | ***ADP 2 µM*** | | |
| CTR (725/330) | 90 (70;94) | 86 (37;93) | <0.001 |
| ASA (2056/1229) | 49 (20;68) | 40 (0;60) | <0.001 |
| CLOP (271/158) | 0 (0;64) | 0 (0;40) | 0.018 |
| DAPT (165/319) | 0 (0;30) | 0 (0;10) | 0.169 |
|  | ***Collagen 2 µg/ml*** | | |
| CTR (725/330) | 92 (89;95) | 93 (90;95) | 0.009 |
| ASA (2056/1223) | 42 (24;68) | 35 (20;57) | <0.001 |
| CLOP (269/157) | 88 (82;90) | 88 (82;91) | 0.568 |
| DAPT (156/287) | 18 (0;40) | 17 (0;28) | 0.242 |
|  | ***Epinephrine 10 µM*** | | |
| CTR (725/330) | 90 (84;94) | 90 (83;95) | 0.732 |
| ASA (2056/1229) | 40 (0;56) | 39 (0;55) | 0.188 |
| CLOP (271/157) | 90 (80;93) | 88 (72;92) | 0.134 |
| DAPT (165/319) | 0 (0;55) | 0 (0;46) | 0.556 |
|  | ***Arachidonic Acid*** | | |
| CTR (658/299) | 92 (90;95) | 93 (90;95) | 0.157 |
| ASA (869/492) | 15 (0;26) | 14 (0;22) | 0.009 |
| CLOP (245/140) | 90 (86;93) | 90 (85;93) | 0.476 |
| DAPT (61/121) | 15 (12;50) | 0 (0;19) | 0.091 |

1. Born GVR. The aggregation of blood platelets by adenosine diphosphate and its reversal. Nature. 1962;194:927-9.
